# Knowledge, attitudes, and practices of seasonal influenza vaccination in healthcare workers, Honduras

**DOI:** 10.1371/journal.pone.0246379

**Published:** 2021-02-04

**Authors:** Zachary J. Madewell, Rafael Chacón-Fuentes, Jorge Jara, Homer Mejía-Santos, Ida-Berenice Molina, Juan Pablo Alvis-Estrada, Maria-Renee Ortiz, Rosa Coello-Licona, Belinda Montejo

**Affiliations:** 1 Centro de Estudios en Salud, Universidad del Valle de Guatemala, Guatemala City, Guatemala; 2 Unidad de Vigilancia de la Salud, Secretaría de Salud de Honduras, Tegucigalpa, Honduras; 3 Programa Ampliado de Inmunizaciones, Secretaría de Salud de Honduras, Tegucigalpa, Honduras; 4 Vigilancia Epidemiológica, Instituto Hondureño de Seguridad Social, Tegucigalpa, Honduras; Waikato Institute of Technology, NEW ZEALAND

## Abstract

**Background:**

Seasonal influenza is a highly contagious vaccine-preventable disease that may cause high morbidity and mortality in susceptible populations. Healthcare workers are a priority group for seasonal influenza vaccination to protect them from contracting influenza and prevent nosocomial transmission to patients. This study aimed to evaluate knowledge, attitudes, and practices (KAP) of seasonal influenza vaccination among healthcare workers in Honduras.

**Method:**

From August 24 to October 21, 2018, we conducted a cross-sectional KAP survey regarding seasonal influenza vaccination to a random sample of healthcare workers who attended patients in hospitals of the Ministry of Health of Honduras (SESAL) and Honduran Social Security Institute (IHSS). We reported frequency distributions of demographics, vaccination KAP, sources of information, and reasons for non-vaccination. We used principal components factor analysis to create knowledge and attitude scores. We used linear regression to analyze associations between demographics and sources of information about the influenza vaccine, and knowledge and attitude scores. We used logistic regression to analyze associations between demographics, sources of information, knowledge scores, and attitude scores, and influenza vaccination.

**Result:**

We surveyed 947 healthcare workers who attended patients in 13 SESAL hospitals and two IHSS hospitals. Only 4.6% of participants knew the seasonal influenza vaccine was composed of inactivated viruses, 94.7% believed vaccination causes flu-like symptoms, and 52.0% were vaccinated for influenza in 2018. Knowledge scores were lower for nursing assistants and other healthcare professionals compared to doctors, and higher for healthcare workers who attended a healthcare facility training (*P*-values≤0.030). Attitude scores were higher for healthcare workers who attended ≥11 patients per day having ≤10 patients per day as reference, self-reported influenza vaccination in previous year, and cited trainings and informal information at the healthcare facility as sources of information for influenza vaccination (*P*-values≤0.030). Factors associated with self-reported vaccination were self-reported influenza vaccination in previous year (aOR: 7.61; 95% CI: 5.24–11.04), attitude score (aOR: 1.14; 95% CI: 1.07–1.21), and worked in a SESAL hospital (aOR: 1.73; 95% CI: 1.12–2.68) having IHSS as reference.

**Conclusion:**

Although influenza vaccination is required by law in Honduras and available for free in public health centers, coverage of healthcare workers in 2018 was half that reported in 2017. Lower coverage may be attributed to misconceptions of vaccination side effects.

## Introduction

Seasonal influenza is a highly contagious vaccine-preventable disease that may cause high morbidity and mortality in susceptible populations. Influenza infection may be mild to severe, causing a myriad of respiratory tract diseases, including pneumonia and acute respiratory distress syndrome. Influenza virus mutates rapidly and its seasonal annual epidemics affect 5–15% of the global population, causing 290,000–650,000 deaths worldwide annually, which is more than all other vaccine-preventable diseases combined [1, 2]. Infected individuals may transmit influenza virus for up to 24 hours before they are symptomatic [3]. Hospitalized patients are particularly susceptible to influenza infections due in part to underlying illnesses. Because of its short incubation period, propensity to mutate, and effective aerosol transmission, influenza virus may cause large hospital outbreaks and closures of entire healthcare facilities [4]. Influenza is also associated with a significant economic burden attributable to direct and indirect healthcare costs [5]. This study focuses on Honduras, where influenza-related mortality, hospitalization, and incidence rates were 0.7 (95% CI: 0.3–1.2), 66.2 (95% CI: 20.0–197.8), and 645.9 (95% CI: 430.9–925.5) per 100,000 people, respectively, in 2017 [6]. Influenza and pneumonia account for 3.7% of all deaths in Honduras [7].

Seasonal influenza vaccination is the most effective strategy for preventing influenza virus infection and its complications [8]. World Health Organization (WHO) Strategic Advisory Group of Experts on Immunization and the Advisory Committee on Immunization Practices of the United States recommend that healthcare workers get annual vaccinations for seasonal influenza [8]. Healthcare workers are a priority group for seasonal influenza vaccination in order to protect them from contracting influenza, prevent nosocomial transmission to vulnerable patients (e.g., children, elderly, immunocompromised), reduce absenteeism caused by seasonal influenza, and maintain healthcare services during influenza epidemics [9]. Vaccine effectiveness is 40–60% among healthy adults in years when vaccine viruses match circulating viruses and influenza-specific antibodies may persist up to 6–8 months [8].

Despite these recommendations, influenza vaccination coverage among healthcare workers varies widely worldwide, ranging from >90% in several Central American countries [10] to <5% in Southeast Asia [11], which is well short of the 80% vaccination rate threshold proposed to reach herd immunity within healthcare facilities for seasonal influenza [12]. Up to 25% of healthcare workers contract influenza annually and many healthcare workers continue to work while sick [13]. Reasons for low influenza vaccination coverage include low perception of risk, fear of adverse side effects, misconceptions regarding vaccine safety and efficacy, and poor knowledge of influenza [14, 15]. Healthcare workers who have unfavorable attitudes, hesitancy, or aversion to vaccination are less likely to recommend vaccination to their patients [16–18]. Direct physician recommendations are one of the most important factors influencing an individual’s decision to get vaccinated [19, 20]. Understanding factors limiting coverage may serve to guide interventions to increase vaccine acceptance among healthcare workers, who are the main facilitators and recommenders of vaccination to patients.

In Honduras, the Expanded Program of Immunization (EPI) of the Ministry of Health (SESAL) was established in 1979 to reduce morbidity and mortality from vaccine-preventable diseases through mass vaccination, epidemiological surveillance, and social participation [21]. EPI is supported by WHO, Pan American Health Organization (PAHO), and United Nations Children’s Fund and delivers >90% of vaccines in Honduras [22]. Under the Vaccine Law of the Republic of Honduras, all residents, including healthcare workers, are legally required to be vaccinated for all vaccine-preventable diseases determined by the SESAL, which includes influenza [23]. Influenza vaccines are available free-of-charge in public health centers and other IHSS healthcare facilities nationwide [24]. The Vaccine Law ensures the budget for vaccines, syringes, and supplies for EPI [23]. EPI’s ambitious vaccination program has led to high coverage rates for vaccine-preventable diseases in Honduras, particularly among high risk groups. In 2017, PAHO reported 100% seasonal influenza vaccination coverage for all healthcare workers, including administrative and support staff [10]. It is important to determine whether this high coverage is truly indicative of practices among healthcare workers in direct contact with patients and to determine what knowledge and attitudes influence healthcare workers’ decisions to get vaccinated. The objectives of this study are therefore to determine knowledge, attitudes, and practices (KAP) regarding seasonal influenza vaccination among healthcare workers who attended patients in Honduras.

## Materials and methods

### Study design

We conducted a cross-sectional KAP survey regarding seasonal influenza vaccinations to a sample of healthcare workers who attended patients in the hospitals of SESAL and Honduran Social Security Institute (IHSS).

### Study setting

Honduras has an area of 112,492 km^2^ and is divided administratively into 18 departments (political subdivisions similar to provinces or states) and 298 municipalities [25]. Honduras has a population of 9,746,000 of which 57.7% reside in urban areas [26]. The life expectancy is 71.3 years (female: 73.0 years; male: 69.6 years) and death rate is 5.3 deaths per 1,000 population [25].

The healthcare system in Honduras consists of a public and private sector. The public sector includes SESAL and IHSS. SESAL provides services to 60% of the population, IHSS serves 12%, and the private sector serves 10% [27, 28]. Approximately 17% of the population does not have access to healthcare services [29]. SESAL, which is administered through 20 health regions (18 departmental and two metropolitan), has seven national hospitals located in Tegucigalpa and San Pedro Sula, six regional hospitals, 16 area hospitals, and 1,606 first-level outpatient facilities [28]. IHSS has two hospitals located in Tegucigalpa and San Pedro Sula and 11 outpatient healthcare facilities [28]. In 2015, SESAL had approximately 2,500 doctors, private sector had 900 doctors, and IHSS had 500 doctors [28]. Healthcare expenditure was 7.6% of the total gross domestic product in 2015 [25]. In 2015, there were 10.1 doctors, 2 professional nurses, and 8.1 auxiliary nurses per 10,000 population, which falls short of the WHO recommendation of 25 doctors and 50 nurses per 10,000 population [30, 31].

### Questionnaire

We adapted a questionnaire from the Centers for Disease Control and Prevention (CDC) influenza survey [32] and previous experiences from another KAP study of healthcare workers in Costa Rica [33]. The questionnaire was modified following an evaluation of technical detail and cultural appropriateness by an anthropologist, technical staff at SESAL, and by the Institutional Review Boards (IRB) of Universidad del Valle de Guatemala (UVG) and Universidad Nacional Autónoma de Honduras (UNAH). We pilot-tested the questionnaire with a group of healthcare workers (medical doctors, nurses, and laboratory personnel) at SESAL in Tegucigalpa four weeks before study implementation. We subsequently modified several questions following feedback provided by the participants. The finalized questionnaire included demographics (age, sex, education, marital status, profession, years in profession, works in multiple healthcare facilities, number of patients attended per day, service network), knowledge and attitudes of influenza vaccination, self-reported influenza vaccination status, sources of information of influenza vaccination, clinical manifestations following vaccination, and reasons for non-vaccination (S1 Questionnaire).

We conducted close-ended surveys from August 24 to October 21, 2018, three months after the launch of the influenza vaccination campaign of Honduras on May 14, 2018. We administered surveys in the hospitals of SESAL and IHSS. Surveys were done by interviews and data collected with tablets, using the Research Data Management Center application (Open Data Kit ODK JAVA). Interviewers were healthcare professionals trained in relevant aspects of influenza vaccination.

### Study population

To calculate the sample size of healthcare workers, we used the lowest administrative vaccination coverage for influenza among healthcare workers in Central American countries reported by Pan American Health Organization (PAHO) in 2015 as a key indicator: 41% [10]. The number of healthcare workers listed for the hospitals of SESAL (9,646 people) [34] and IHSS (2,330 people) [35] was used as the reference population. We used a design effect of two, corresponding to the two stages of sampling described below. We also used a high replacement rate of 25% because of previous experiences from another KAP study of healthcare workers in Costa Rica [33]. Using 5% accuracy and a 95% confidence interval, we calculated a sample size of 954 healthcare workers (S1 File).

**Fig 1:**
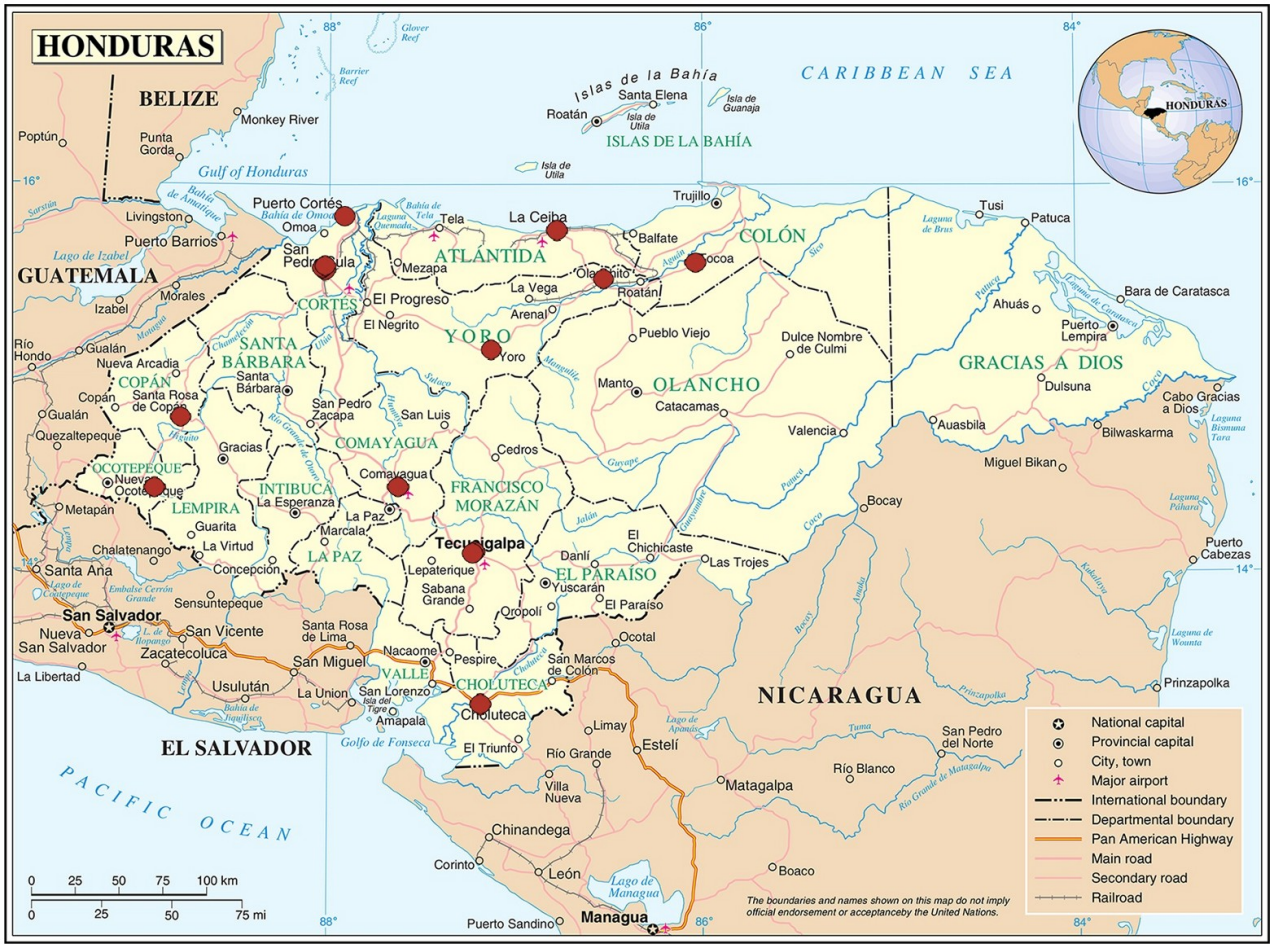
Locations of 15 healthcare facilities from Ministry of Health of Honduras and Honduran Social Security Institute, study of knowledge, attitudes and practices of seasonal influenza vaccination, healthcare workers, Honduras, 2018. Source: This map is a public domain file provided by the Central Intelligence Agency’s World Factbook, https://www.cia.gov/library/publications/the-world-factbook/.

We used separate probabilistic, two-stage, stratified and conglomerate sampling to select samples of healthcare workers who attended patients in SESAL and IHSS hospitals. Stratification was based on hospital locations (West, Northeast, Central). In stage one, we identified conglomerates (hospitals) in each stratum by probability proportional to the number of healthcare workers who attended patients in each healthcare facility. In stage two, we identified healthcare workers in each selected conglomerate by simple random sampling within each group of healthcare professionals. The groups were doctors (general practitioners or medical specialists), nurses (auxiliary or professional), and other healthcare workers in direct contact with patients (e.g., dentists, psychologists, social workers, radiology technicians, laboratory staff, cleaning staff, customer service staff, others). Healthcare facilities were located in nine of the 18 departments of Honduras (Fig 1).

We invited healthcare workers ages ≥18 years who attended patients in SESAL or IHSS hospitals from August 24 to October 21, 2018 to participate. We excluded administrative/support staff who did not attend patients directly.

### Ethics statement

This study was approved by the Research Ethics Committee of UVG (Protocol number 173-10-2017), Bioethics Committee of UNAH (study code 2018011), and Teaching and Research Department of the IHSS. We obtained written informed consent for all participants.

### Statistical analysis

We reported frequency distributions of demographic variables (age group, sex, education, marital status, profession, years in profession, works in multiple healthcare facilities, number of patients attended per day, service network, health system, self-reported current vaccination status [2018] and vaccination status in previous year [2017]) for participants included in KAP surveys. We reported frequency distributions and 95% confidence intervals (CI) for knowledge and attitudes of influenza virus, transmission, and vaccination; sources of information about influenza vaccination; clinical manifestations seven days after vaccination; and reasons for non-vaccination. For responses regarding knowledge and attitudes of seasonal influenza vaccination, we reported proportions who fully agreed and agreed but with doubt and excluded participants who did not respond. For healthcare workers who obtained influenza vaccination information from mass media, we reported proportions and 95% CIs of those who fully and partially trusted the indicated source.

We used principal components factor analysis (PCA) to create a knowledge score based on seven variables and an attitude score based on nine variables for all healthcare workers (S1 Table). First, we assigned scores ranging from 0–3 for each variable (3: strongly agree; 2: slightly agree; 1: slightly disagree; 0: strongly disagree). People who did not know or did not respond to a knowledge or attitude question were assigned a score of 0 for that question. The resultant compound factor for knowledge variables included four variables that accounted for 21.8% of the variability in the data: knowledge that influenza may be transmitted from birds/pigs to people, people may contract influenza multiple times, influenza may be spread via contaminated hands, and healthcare workers may transmit influenza to patients (S2 Table). The resultant compound factor for attitude variables included eight variables that accounted for 39.0% of the variability in the data: belief that vaccination is effective at preventing influenza, lowers risk of hospitalization/death, decreases days of illness, and protects patients; healthcare personnel should get vaccinated every year; would get vaccinated if offered vaccine at home or at work; and recommends vaccination to family and friends. Subsequent factors explained little variability. Therefore, only the first factor was retained from each PCA, which we termed “knowledge score” and “attitude score.” These variables were then weighted against their eigenvector coefficients. Knowledge and attitude scores ranged from 0–7 and 0–16, respectively, with higher scores indicating greater knowledge and more positive attitudes.

We reported means and standard deviations for knowledge and attitude scores by demographic variables and sources of information about seasonal influenza vaccination. T-tests and analysis of variance (ANOVA) were used to examine associations between demographics and sources of information, and knowledge and attitude scores. Bonferroni was used for post-hoc ANOVA comparisons. We used Pearson correlation to evaluate associations between knowledge and attitude scores.

We used linear regression to analyze associations between demographics and sources of information about the influenza vaccine, and knowledge and attitude scores. Knowledge score was also included as an exposure variable for regression analysis of attitude score. Statistical significance was determined using the Wald F-test. We used logistic regression to analyze associations between demographics, sources of information, and knowledge and attitude scores, and self-reported current influenza vaccination. Significance was evaluated through the Wald Chi-square test. Variables found to be significant at *P*<0.20 from unadjusted analyses were included in manual forward step-wise multivariable linear and logistic regression models to evaluate associations with outcomes (knowledge scores, attitude scores, and influenza vaccination). Variables with the smallest *P-*value from unadjusted analyses were added one at a time to the forward step-wise regression models and removed at a *P<*0.20 significance level. Values of *P*<0.05 were considered statistically significant. These analyses excluded participants who did not know if they were vaccinated for influenza in 2017 or 2018. We used tolerance values to assess collinearity among all independent variables. Hosmer-Lemeshow was used to assess goodness-of-fit of the final adjusted logistic regression model. We used SAS V.9.4 (SAS Institute, Inc., Cary, North Carolina) for all analyses.

## Results

### Sample characteristics

We surveyed 947 healthcare workers who attended patients in 13 SESAL hospitals and two IHSS hospitals (Fig 1). The median age was 42 years (interquartile range: 35–51 years) and median years in profession was 15 years (interquartile range: 6–23 years). Of all participants, 77.5% were female; 72.2% were doctors, nursing professionals, or nursing assistants; 43.7% were married; and 84.4% worked in SESAL hospitals (Table 1). Of 184 doctors, 70% were specialists.

**Table 1:** Demographics and influenza vaccination coverage of 947 healthcare workers, Honduras, August 24 to October 21, 2018.

Characteristic	n (%)
Age (in years)	
20–30	150 (15.8)
31–40	267 (28.2)
≥41	530 (56.0)
Female sex	734 (77.5)
Educational attainment	
≤Middle school	176 (18.6)
High school	258 (27.2)
University	371 (39.2)
Postgraduate, masters, doctorate	142 (15.0)
Marital status	
Single	351 (37.1)
Married	414 (43.7)
Accompanied	132 (13.9)
Other	50 (5.3)
Profession	
Doctor	184 (19.4)
Nursing professional	141 (14.9)
Nursing assistant	359 (37.9)
Other healthcare profession	263 (27.8)
Years in profession	
≤10	388 (41.0)
>10	559 (59.0)
Works in multiple healthcare facilities	282 (29.8)
Number of patients attended per day	
≤10	238 (25.1)
11–20	221 (23.3)
21–30	210 (22.2)
≥31	278 (29.4)
Service network	
Central	444 (46.9)
North	396 (41.8)
West	69 (7.3)
South	38 (4.0)
Health system	
Ministry of Health of Honduras	799 (84.4)
Honduran Social Security Institute	148 (15.6)
Self-reported influenza vaccination in previous year (n = 935)^a^	633 (67.7)
Self-reported current influenza vaccination (n = 945)^b^	491 (52.0)

^a^ Excluded 12 who did not know vaccination status.

^b^ Excluded 2 who did not know vaccination status.

### Sources of information

Of 947 healthcare workers, 346 learned about the influenza vaccine from informal information at the healthcare facility (36.5%; 95% CI: 33.5–39.6%) and 358 from self-teaching (37.8%; 95% CI: 34.7–40.9%) (Table 2). Of the latter 358 participants, 292 learned from scientific literature on the internet (81.6%; 95% CI: 77.5–85.6%). Of 286 participants who cited mass media as their source of information for seasonal influenza vaccination, the most frequently cited source was television (78.0%; 95% CI: 73.1–82.8%) (S3 Table). However, the most trusted sources were PAHO (57.4% fully trusted; 95% CI: 51.6–63.2%) and CDC (48.2% fully trusted; 95% CI: 42.4–54.1%).

**Table 2:** Sources of information about influenza vaccination, healthcare workers, Honduras, August 24 to October 21, 2018 (n = 947).

Source of information	n	% (95% CI)
Informal information from the healthcare facility	346	36.5 (33.5–39.6)
Mass media	286	30.2 (27.3–33.1)
Coworkers or peers	239	25.2 (22.5–28.0)
Training in the healthcare facility	215	22.7 (20.0–25.4)
Doctor or nurse at healthcare facility	133	14.0 (11.8–16.3)
Family or friends	63	6.7 (5.1–8.2)
Brochures or posters	34	3.6 (2.4–4.8)
Medical consultation	26	2.7 (1.7–3.8)
Vocational training	7	0.7 (0.2–1.3)
*Self-taught (n = 358)*	358	37.8 (34.7–40.9)
Scientific literature published on the internet	292	81.6 (77.5–85.6)
Medical books	104	29.1 (24.3–33.8)
Printed scientific journals	70	19.6 (15.4–23.7)
Personal experience from the clinic	71	19.8 (15.7–24.0)

CI: confidence interval.

### Knowledge of influenza vaccination

Although 95.0% of healthcare workers knew they may transmit influenza to their patients (95% CI: 93.6–96.4%), only 60.8% recognized that influenza may be transmitted from birds or pigs to people (95% CI: 57.5–64.2%) (Table 3). Furthermore, only 4.6% of healthcare workers knew the 2018 influenza vaccine was composed of inactivated viruses (95% CI: 3.3–6.0%). Unadjusted analyses between demographics and sources of information, and knowledge scores are reported in S4 and S5 Tables.

**Table 3:** Knowledge of influenza vaccine, healthcare workers, Honduras, August 24 to October 21, 2018.

	Participants^a^	Fully agree		Agree but with doubt	
Knowledge	n	n	% (95% CI)	n	% (95% CI)
People may spread influenza even without symptoms	913	696	76.2 (73.5–79.0)	85	9.3 (7.4–11.2)
Influenza may be transmitted from birds or pigs to people	807	491	60.8 (57.5–64.2)	112	13.9 (11.5–16.3)
People may contract influenza even if they have previously contracted influenza	902	762	84.5 (82.1–86.8)	76	8.4 (6.6–10.2)
Influenza may be spread by touching one’s mouth or nose with contaminated hands	932	780	83.7 (81.3–86.1)	72	7.7 (6.0–9.4)
Healthcare workers may transmit influenza to their patients	941	894	95.0 (93.6–96.4)	21	2.2 (0.5–3.2)
Received adequate information to decide whether to get vaccinated	944	331	35.1 (32.0–38.1)	132	14.0 (11.8–16.2)
The influenza vaccine is composed of inactivated viruses	947	44	4.6 (3.3–6.0)	0	–
Knowledge score^b^: *mean =* 5.91; *standard deviation*: 1.18					

CI: confidence interval.

^a^ Excluded healthcare workers who did not respond.

^b^ Knowledge score was derived from principal components analysis and included: knowledge that influenza may be transmitted from birds/pigs to people, people may contract influenza multiple times, influenza may be spread via contaminated hands, and healthcare workers may transmit influenza to patients; range: 0–7.

The final model for knowledge score included profession and learned about influenza vaccinations from healthcare facility trainings. Adjusting for the other variable in the model, knowledge scores were significantly lower for nursing assistants (β: -0.44, *P*<0.001) and other healthcare professionals (β: -0.37, *P*<0.001) compared to doctors, and higher for healthcare workers who attended a healthcare facility training (β: 0.20, *P* = 0.030) (S6 Table). Tolerance values were >0.99, so there was no evidence of collinearity.

### Attitudes towards influenza vaccination

Almost all participants believed healthcare workers should be vaccinated for seasonal influenza every year (95.2%; 95% CI: 93.8–96.6%), but 94.7% believed the vaccine causes flu-like symptoms (95% CI: 93.3–96.1%) (Table 4). Knowledge score was associated with attitude score (*r =* 0.08; *P =* 0.016). Unadjusted analyses between demographics and sources of information, and attitude scores are reported in S4 and S5 Tables.

**Table 4:** Attitudes towards influenza vaccine, healthcare workers, Honduras, August 24 to October 21, 2018.

	Participants^a^	Fully agree		Agree but with doubt	
Attitude	n	n	% (95% CI)	n	% (95% CI)
The vaccine is effective at preventing influenza	907	553	61.0 (57.8–64.2)	189	20.8 (18.2–23.5)
The vaccine lowers the risk of hospitalization and death	929	762	82.0 (79.5–84.5)	115	12.4 (10.3–14.5)
The vaccine may decrease the days of illness from influenza	893	599	67.1 (64.0–70.2)	139	15.6 (13.2–17.9)
Vaccinating healthcare personnel protects patients	942	817	86.7 (84.6–88.9)	68	7.2 (5.6–8.9)
Healthcare personnel should get vaccinated for influenza every year	920	876	95.2 (93.8–96.6)	0	–
The influenza vaccine causes flu-like symptoms	925	876	94.7 (93.3–96.1)	0	–
Would get vaccinated for influenza if offered the vaccine at work	927	775	83.6 (81.2–86.0)	0	–
Would get vaccinated for influenza if offered the vaccine at home	932	812	87.1 (85.0–89.3)	0	–
Recommends the influenza vaccine to family and friends	930	845	90.9 (89.0–92.7)	0	–
Attitude score^b^: *mean =* 13.32; *standard deviation*: 3.40					

CI: confidence interval.

^a^ Excluded healthcare workers who did not respond.

^b^ Attitude score was derived from principal components analysis and included: belief that vaccination is effective at preventing influenza, lowers risk of hospitalization/death, decreases days of illness, and protects patients; healthcare personnel should get vaccinated every year; would get vaccinated if offered vaccine at home or at work; and recommends vaccination to family and friends; range: 0–16.

The final model for attitude score included years in profession, number of patients attended per day, self-reported influenza vaccination in previous year, knowledge score, and learned about influenza vaccinations from informal information at healthcare facility, healthcare facility trainings, and mass media. Adjusting for the other variables in the model, attitude scores were significantly higher for healthcare workers who attended >30 (β: 0.99, *P*<0.001), 21–30 (β: 0.61, *P* = 0.035), and 11–20 (β: 0.89, *P* = 0.002) patients per day having ≤10 as reference; self-reported influenza vaccination in previous year (β: 2.97, *P*<0.001); and cited trainings (β: 1.11, *P*<0.001) and informal information at the healthcare facility (β: 0.44, *P* = 0.030) as sources of information for influenza vaccination (S6 Table). Tolerance values were >0.98.

### Influenza vaccination

Nine hundred forty-five healthcare workers of 947 knew their vaccination status, of whom 491 self-reported vaccination for seasonal influenza in 2018 (52.0%; 95% CI: 48.8–55.1%) (Table 1). Influenza vaccination coverage ranged from 24.7–87.9% between hospitals, whereas the proportion of healthcare workers who would get vaccinated for influenza if offered vaccination at work ranged from 69.8–97.0% between hospitals (S1 Fig).

The final model for self-reported current influenza vaccination included education, marital status, years in profession, service network, health system, self-reported influenza vaccination in previous year, attitude score, and learned about influenza vaccinations from healthcare facility trainings and coworkers or peers (Table 5). Adjusting for the other variables in the model, the odds of self-reported current influenza vaccination were 7.61 times higher for healthcare workers who self-reported influenza vaccination in previous year (95% CI: 5.24–11.04), were 1.73 times higher for those who worked in a SESAL hospital having IHSS as reference (95% CI: 1.12–2.68), and increased by a factor of 1.14 for every one-unit increase in attitude score (95% CI: 1.07–1.21). The Hosmer-Lemeshow goodness-of-fit test demonstrated the model fit was adequate (p = 0.86). Tolerance values were >0.78.

**Table 5:** Associations between demographics, sources of information, knowledge^a^ and attitude^b^ scores, and influenza vaccination, healthcare workers, Honduras, August 24 to October 21, 2018 (n = 933)^a^.

Variable	OR (95% CI)	*P*-value	aOR^c^ (95% CI)	*P*-value
Age in years (Ref: ≥41)		0.816		–
20–30	1.12 (0.78–1.62)		–	
31–40	1.05 (0.78–1.41)		–	
Female sex (Ref: male)	1.10 (0.81–1.50)	0.532	–	
Education (Ref: ≤middle school)		0.031		0.081
High school	0.94 (0.64–1.39)		0.88 (0.56–1.39)	
University	1.37 (0.95–1.97)		1.41 (0.91–2.17)	
Postgraduate, masters, doctorate	0.83 (0.53–1.29)		0.94 (0.55–1.62)	
Marital status (Ref: married)		0.040		0.099
Single	0.84 (0.63–1.12)		0.77 (0.55–1.09)	
Accompanied	1.42 (0.95–2.12)		1.36 (0.84–2.21)	
Other	0.65 (0.36–1.18)		0.74 (0.37–1.49)	
Profession (Ref: doctor)		0.178		–
Nursing professional	1.45 (0.93–2.27)		–	
Nursing assistant	0.93 (0.65–1.33)		–	
Other	1.02 (0.70–1.49)		–	
≤10 years in profession (Ref: >10 years)	1.32 (1.02–1.72)	0.038	1.27 (0.92–1.74)	0.143
Works in multiple healthcare facilities (Ref: no)	0.85 (0.64–1.12)	0.245	–	
Number of patients attended per day (Ref: ≤10)		0.043		–
11–20	1.21 (0.83–1.75)		–	
21–30	1.54 (1.05–2.24)		–	
>30	1.57 (1.11–2.24)		–	
Service network (Ref: Central)		0.032		0.149
North	0.87 (0.66–1.14)		0.82 (0.59–1.13)	
West	1.33 (0.79–2.23)		0.93 (0.51–1.69)	
South	2.42 (1.14–5.13)		2.26 (0.90–5.65)	
Ministry of Health of Honduras health system (Ref: Honduran Social Security Institute)	1.90 (1.32–2.72)	<0.001	1.73 (1.12–2.68)	0.013
Source of information (Ref: no)				
Family or friends	1.00 (0.60–1.67)	0.994	–	–
Coworkers or peers	0.75 (0.55–1.00)	0.052	0.78 (0.55–1.10)	0.160
Information informally provided in healthcare facility	1.19 (0.91–1.56)	0.201	–	–
Training in healthcare facility	1.76 (1.29–2.40)	<0.001	1.34 (0.93–1.92)	0.121
Doctor or nurse at healthcare facility	0.82 (0.57–1.18)	0.285	–	–
Medical consultation	0.52 (0.23–1.18)	0.118	–	–
Brochures or posters	0.93 (0.47–1.85)	0.843	–	–
Vocational training	1.25 (0.28–5.60)	0.773	–	–
Self-taught	0.83 (0.64–1.08)	0.163	–	–
From mass media	0.70 (0.53–0.93)	0.014	–	–
Vaccinated for influenza in previous year (Ref: no)	10.19 (7.24–14.35)	<0.001	7.61 (5.24–11.04)	<0.001
Knowledge score^a^ (1-unit increase)	1.06 (0.95–1.18)	0.287	–	–
Attitude score^b^ (1-unit increase)	1.27 (1.21–1.34)	<0.001	1.14 (1.07–1.21)	<0.001

Ref: reference; OR: odds ratio; aOR: adjusted odds ratio; CI: confidence interval.

^a^ Knowledge score was derived from principal components analysis and included: knowledge that influenza may be transmitted from birds/pigs to people, people may contract influenza multiple times, influenza may be spread via contaminated hands, and healthcare workers may transmit influenza to patients; range: 0–7.

^b^ Attitude score was derived from principal components analysis and included: belief that vaccination is effective at preventing influenza, lowers risk of hospitalization/death, decreases days of illness, and protects patients; healthcare personnel should get vaccinated every year; would get vaccinated if offered vaccine at home or at work; and recommends vaccination to family and friends; range: 0–16.

^c^ Adjusted for the other variables listed in the model.

^d^ Excluded 12 who did not know vaccination status.

^e^ Excluded 2 who did not know vaccination status.

Of 491 healthcare workers who were vaccinated for influenza, 187 (37.9%; 95% CI: 33.6–42.2%) reported mild or moderate untoward reactions after vaccination, including vaccination site pain, flu-like symptoms, and general discomfort (S7 Table).

### Reasons for non-vaccination

Of 454 healthcare workers who were not vaccinated for seasonal influenza, 207 cited access limitations (e.g., time constraints, not being offered vaccine) (45.6%; 95% CI: 41.0–50.2%) and 189 cited fear of adverse effects (41.6%; 95% CI: 37.1–46.2%) as reasons for non-vaccination (Table 6).

**Table 6:** Reasons for not receiving influenza vaccination, healthcare workers (n = 454), Honduras, August 24 to October 21, 2018.

Reasons	n	% (95% CI)
*Fear of adverse effects*	189	41.6 (37.1–46.2)
Fear of side effects	140	30.8 (26.6–35.1)
Fear of contracting influenza	77	17.0 (13.5–20.4)
Fear of injection pain	31	6.8 (4.5–9.2)
Was breastfeeding	4	0.8 (0–1.7)
Was pregnant	1	0.2 (0–0.7)
*Perception of lack of utility of vaccination*	75	16.5 (13.1–19.9)
No confidence in current vaccines	30	6.6 (4.3–8.9)
Believes the vaccine is not effective	28	6.2 (3.9–8.4)
Influenza does not cause serious illness	12	2.6 (1.2–4.1)
Not in contact with patients who have influenza	7	1.5 (0.4–2.7)
No confidence in vaccine cold chain	6	1.3 (0.3–2.4)
The vaccine does not prevent colleagues from contracting influenza	2	0.4 (0–1.1)
*Limited access to vaccines*	207	45.6 (41.0–50.2)
Too busy to get vaccinated	120	26.4 (22.4–30.5)
Was not offered the vaccine	98	21.6 (17.8–25.4)
Not informed to get vaccinated	11	2.4 (1.0–3.8)
Unaware of where to get vaccinated	10	2.2 (0.8–3.6)
Vaccine is too expensive	1	0.2 (0–0.7)
*Social influence*	5	1.1 (0.1–2.1)
Vaccine not accepted by peers	2	0.4 (0–1.1)
Relatives said not to get vaccinated	2	0.4 (0–1.1)
Friends said not to get vaccinated	2	0.4 (0–1.1)
Boss did not give permission to get vaccinated	1	0.2 (0–0.7)

CI: confidence interval.

Composite subheadings (e.g., fear of adverse effects) included at least one positive response for one of the listed reasons.

## Discussion

Influenza vaccination coverage in a sample of healthcare workers who attended patients in hospitals was 52.0%, which is almost half the coverage reported by PAHO for healthcare workers in Honduras in 2017 (100%) [10]. Coverage was also lower than that of healthcare workers in Panama (92%), Costa Rica (88%), Guatemala (74%) and El Salvador in 2018 (61%), which may be attributed to differences in vaccination schemes, implementation frames, targeted healthcare worker groups, vaccine availability, communication activities, and previous experiences with influenza [10]. However, this study only included healthcare workers who attended patients, whereas the PAHO figures were for all healthcare workers, including administrative staff who were not in contact with patients.

Although influenza vaccination is required by law in Honduras and is available for free in public health centers and other healthcare facilities nationwide, there are no penalties for unvaccinated healthcare workers [23, 24, 36]. Additionally, although mandatory influenza vaccination policies among healthcare workers have been demonstrated to increase vaccination rates in other settings, enforcement remains challenging [9, 37]. Requiring healthcare workers who decline vaccination to wear a mask while in contact with patients in the healthcare facility has been demonstrated to be a cost-effective strategy at increasing influenza vaccination coverage [9, 38]. This may be due in part to the inconvenience and stigma associated with wearing masks in the healthcare facility [38]. Other strategies including mobile vaccination teams, walk-in vaccinations, on-site vaccinations, and use of declination forms have had moderate success in improving influenza vaccination rates [9].

Low seasonal influenza vaccination coverage among healthcare workers in 2018 may be attributed to misconceptions of influenza virus and vaccine. The main knowledge gap was not knowing the vaccine was composed of inactive viruses or segments of viruses that are noninfectious. These results were supported by the finding that most participants believed the vaccine may cause influenza-like symptoms. Furthermore, some of the vaccinated participants mentioned they had flu-like symptoms within one week of receiving the influenza vaccine. Among unvaccinated participants, the main reason for declining vaccination was fear of side effects and of contracting influenza. These findings are consistent with other studies [14, 15, 39]. Anti-vaccination conspiracy theories may play some role by spreading false information about vaccine side effects and understating the risk of influenza [16]. Lower coverage in 2018 may in part also be attributed to a reduced influenza season in Honduras in 2017 compared to other years (e.g., 2014) [40], which could have affected perceptions of the severity of influenza and necessity of vaccination. Alternatively, it is conceivable that the large influenza outbreak in Honduras in 2018 affected perceptions of the efficacy of vaccination [40].

Other reasons for non-vaccination were busy schedules and not being offered the vaccine, which is consistent with other studies [39, 41]. In Honduras, healthcare facilities are advised to maintain the Healthcare Workers Vaccination Listings (LIVATS), which shows vaccination statuses for healthcare workers for all vaccine-preventable diseases. In addition to healthcare workers, LIVATS may include students and volunteers and should indicate whether healthcare workers accepted or rejected vaccination.

The finding that healthcare workers in SESAL were nearly twice as likely to be vaccinated as those in IHSS may be attributed in part to SESAL’s EPI, which set a goal of vaccinating >560,000 Hondurans including 30,000 healthcare workers in 2018 [42].

Despite low influenza vaccine coverage found in healthcare workers, 95% of participants agreed that healthcare workers should be vaccinated annually for influenza and most recommended the vaccine to friends and family. Furthermore, four-fifths of participants would be vaccinated if vaccinations were easily accessible, suggesting healthcare workers are willing to get vaccinated despite fears of adverse effects, which is consistent with a study of nurses in the United States [43]. Offering incentives for vaccination, increased advertising campaigns, and offering a choice of intranasal or injectable vaccines may improve workplace vaccination rates [44].

Many healthcare workers learned about influenza vaccinations from scientific literature on the internet or medical textbooks, which contrasts other studies that cited television and social media as primary sources of information [45, 46]. Informal information at the healthcare facility was associated with more positive attitudes towards influenza vaccination, such as the Weekly Bulletin of SESAL. The bulletin may emphasize that the current influenza vaccine is composed of inactive viruses and scientific evidence of the safety and benefits of vaccination.

Knowledge and attitude scores were higher for healthcare workers who learned about influenza vaccination from formal trainings at healthcare facilities, which is in accord with other studies [47, 48]. As data were collected three months after the launch of influenza vaccination campaigns, this may be attributed in part to education given to healthcare providers at the onset of the campaign. Knowledge scores were also higher for doctors compared to nursing assistants and other healthcare professionals, but knowledge scores were not associated with current vaccination. Knowledge scores in this study primarily consisted of correlates of influenza transmissibility, rather than personal susceptibility, health risk, and economic consequences. Organizing trainings at least one month before onset of the vaccination campaign, including all healthcare workers in trainings, increasing the frequency of educational activities (e.g., during times of low virus circulation), and emphasizing the risk of nosocomial transmission from healthcare workers to patients may also increase coverage.

The finding that vaccination in the previous year had the strongest association with current vaccination is supported by other studies [49, 50]. Higher attitude scores were concomitantly associated with greater vaccination. If healthcare workers have positive initial vaccination experiences, they may be more likely to seek vaccination in following years [51], and subsequently recommend vaccines to their patients [52]. In addition to increasing vaccination coverage in the short term, robust influenza vaccination campaigns may facilitate vaccination the following year [51].

Attending >30 patients per week was associated with more positive attitudes towards influenza vaccination. This is consistent with a study in Japan that found the number of patients seen per day was associated with both intention to be vaccinated for H1N1 and intention to recommend the vaccine to patients [53]. This finding may suggest healthcare workers who see more patients have a higher perception of risk of contracting influenza.

Demographics found to be associated with influenza vaccination among healthcare workers in other studies including physician profession, older age, years of work in healthcare sector, and male gender, were not associated with vaccination in this study [9, 54].

This study had several limitations. First, this study focused on healthcare workers who attended patients in hospitals and may not be generalizable to healthcare workers in other clinical settings. For example, it is unknown whether healthcare workers in primary care clinics had different beliefs and access to vaccines than those in hospitals. Second, this was a cross-sectional study, so we could not establish cause-and-effect relationships between predictor variables and vaccination behavior. Third, influenza vaccinations were self-reported, but other studies have demonstrated strong concordance between self-reported influenza vaccination status and vaccination status reported in medical records [55, 56]. Fourth, there may have been social desirability bias in self-reported KAP of influenza virus and vaccinations. Fifth, there may have been recall bias if there were differences in recall or reporting (e.g., attended training in healthcare facility) between vaccinated and unvaccinated participants. Sixth, there may have been response bias if vaccinated healthcare workers were more inclined to participate. Seventh, the UVG IRB required exclusion of participants who did not have authorization from their institution to participate and administrative/support staff who did not attend patients directly. However, all selected healthcare facilities authorized participation for all of their personnel, therefore this did not contribute to selection bias. Notwithstanding these limitations, our study included a large sample of healthcare workers in both SESAL and IHSS. To our knowledge, this is the first KAP study of seasonal influenza vaccination among healthcare workers in Central America.

Knowledge and attitudes of seasonal influenza vaccination among healthcare workers in Honduras were favorable, but most participants believed the vaccine was composed of live viruses and could cause disease. Factors associated with current vaccination included vaccination in previous year and more positive attitudes. Healthcare authorities in Honduras should encourage all healthcare workers to get vaccinated in compliance with the law and should consider the level of influenza vaccination coverage as a component of a patient safety quality program.

## Supporting information

S1 Table Scores assigned to knowledge and attitude variables for principal components factor analysis, healthcare workers, Honduras, 2018. (DOCX)

S2 Table Principal components factor analysis of knowledge and attitude variables, healthcare workers, Honduras, 2018. (DOCX)

S3 Table Types of media for healthcare workers who cited mass media as a source of information about influenza vaccination, Honduras, 2018. (DOCX)

S4 Table Knowledge and attitude scores for demographics and influenza vaccination status, healthcare workers, Honduras, 2018. (DOCX)

S5 Table Knowledge and attitude scores for sources of information about influenza vaccination, healthcare workers, Honduras, 2018. (DOCX)

S6 Table Associations between demographics and sources of information, and knowledge and attitude scores, healthcare workers, Honduras, 2018. (DOCX)

S7 Table Clinical manifestations seven days after vaccination, healthcare workers, Honduras, 2018. (DOCX)

S1 Fig Seasonal influenza vaccination coverage among 945 healthcare workers, and proportion who would get vaccinated if offered the vaccine at work by hospital, Honduras, 2018. This figure excluded two healthcare workers who did not know their vaccination status.
(DOCX)

S1 Questionnaire (DOCX)

S1 File Equation used to obtain sample sizes for surveys of healthcare workers. (DOCX)
