# Supplementary material for: Knowledge, attitudes, and practices of seasonal influenza vaccination in healthcare workers, Honduras
Source: PLoS One. 2021 Feb 4;16(2):e0246379. doi: 10.1371/journal.pone.0246379 (PMC7861374; doi:10.1371/journal.pone.0246379)
Supplement: S1 Table — (DOCX) [file pone.0246379.s001.docx]

| **S1 Table. Scores assigned to knowledge and attitude variables for principal components factor analysis, healthcare workers, Honduras, 2018** | | | | |
| --- | --- | --- | --- | --- |
|  | Strongly Agree | Slightly agree | Slightly disagree | Strongly disagree |
| *Knowledge of influenza virus, transmission, and vaccine* |  |  |  |  |
| People may spread influenza even without symptoms | 3 | 2 | 1 | 0 |
| Influenza may be transmitted from birds or pigs to people | 3 | 2 | 1 | 0 |
| People may contract influenza even if they have previously contracted influenza | 3 | 2 | 1 | 0 |
| Influenza may be spread by touching one's mouth or nose with contaminated hands | 3 | 2 | 1 | 0 |
| Healthcare workers may transmit influenza to their patients | 3 | 2 | 1 | 0 |
| Received adequate information to decide whether to get vaccinated | 3 | 2 | 1 | 0 |
| The influenza vaccine is composed of inactivated viruses^a^ | 3 | – | – | 0 |
| *Attitudes towards the influenza vaccine* |  |  |  |  |
| The vaccine is effective at preventing influenza | 3 | 2 | 1 | 0 |
| The vaccine lowers the risk of hospitalization and death | 3 | 2 | 1 | 0 |
| The vaccine may decrease the days of illness from influenza | 3 | 2 | 1 | 0 |
| Vaccinating healthcare personnel protects patients | 3 | 2 | 1 | 0 |
| Healthcare personnel should get vaccinated for influenza every year^a^ | 3 | – | – | 0 |
| The influenza vaccine causes flu-like symptoms^a^ | 0 | – | – | 3 |
| Would get vaccinated for influenza if offered the vaccine at work^a^ | 3 | – | – | 0 |
| Would get vaccinated for influenza if offered the vaccine at home^a^ | 3 | – | – | 0 |
| Recommends the influenza vaccine to family and friends^a^ | 3 | – | – | 0 |
| ^a^ The responses for these questions were limited to 'agree' or 'disagree', for which we assigned scores of 3 or 0 | | | | |
