# Supplementary material for: Knowledge, attitudes, and practices of seasonal influenza vaccination in healthcare workers, Honduras
Source: PLoS One. 2021 Feb 4;16(2):e0246379. doi: 10.1371/journal.pone.0246379 (PMC7861374; doi:10.1371/journal.pone.0246379)
Supplement: S2 Table — (DOCX) [file pone.0246379.s002.docx]

| **S2 Table. Principal components factor analysis of knowledge and attitude variables, healthcare workers, Honduras, 2018 (n=947)** | |
| --- | --- |
| Characteristic | Factor pattern |
| **Knowledge score** |  |
| Influenza may be transmitted from birds or pigs to people | 0.43 |
| People may contract influenza even if they have previously contracted influenza | 0.54 |
| Influenza may be spread by touching one's mouth or nose with contaminated hands | 0.64 |
| Healthcare workers may transmit influenza to their patients | 0.71 |
| *Eigenvalue* | 1.38 |
| **Attitude score** |  |
| The vaccine is effective at preventing influenza | 0.67 |
| The vaccine lowers the risk of hospitalization and death | 0.73 |
| The vaccine may decrease the days of illness from influenza | 0.50 |
| Vaccinating healthcare personnel protects patients | 0.60 |
| Healthcare personnel should get vaccinated for influenza every year | 0.50 |
| Would get vaccinated for influenza if offered the vaccine at work | 0.72 |
| Would get vaccinated for influenza if offered the vaccine at home | 0.74 |
| Recommends the influenza vaccine to family and friends | 0.76 |
| *Eigenvalue* | 3.50 |
