# Supplementary material for: Knowledge, attitudes, and practices of seasonal influenza vaccination in healthcare workers, Honduras
Source: PLoS One. 2021 Feb 4;16(2):e0246379. doi: 10.1371/journal.pone.0246379 (PMC7861374; doi:10.1371/journal.pone.0246379)
Supplement: S3 Table — (DOCX) [file pone.0246379.s003.docx]

| **S3 Table. Types of media for healthcare workers who cited mass media as a source of information about influenza vaccination, Honduras, 2018 (n = 286)** | | | | |
| --- | --- | --- | --- | --- |
|  | Trust fully | | Trust partially | |
| Media | n | % (95% CI) | n | % (95% CI) |
| Social networks (e.g., Facebook, Twitter) | 16 | 5.6 (2.9–8.3) | 87 | 30.6 (25.2–36.0) |
| Radio | 46 | 16.2 (11.9–20.5) | 128 | 45.1 (39.2–50.9) |
| Television | 80 | 28.2 (22.9–33.4) | 143 | 50.4 (44.5–56.2) |
| Newspapers | 50 | 17.6 (13.1–22.1) | 121 | 42.6 (36.8–48.4) |
| Medical insurance | 41 | 14.4 (10.3–18.5) | 78 | 27.5 (22.2–32.7) |
| Professional medical associations | 117 | 41.2 (35.4–47.0) | 65 | 22.9 (18.0–27.8) |
| PAHO / WHO | 163 | 57.4 (51.6–63.2) | 40 | 14.1 (10.0–18.2) |
| CDC | 137 | 48.2 (42.4–54.1) | 39 | 13.7 (9.7–17.8) |
| Pharmaceutical companies | 31 | 10.9 (7.3–14.6) | 56 | 19.7 (15.1–24.4) |
| CI: confidence interval; PAHO: Pan American Health Organization; WHO: World Health Organization; CDC: Centers for Disease Control and Prevention | | | | |
