# Supplementary material for: Knowledge, attitudes, and practices of seasonal influenza vaccination in healthcare workers, Honduras
Source: PLoS One. 2021 Feb 4;16(2):e0246379. doi: 10.1371/journal.pone.0246379 (PMC7861374; doi:10.1371/journal.pone.0246379)
Supplement: S4 Table — (DOCX) [file pone.0246379.s004.docx]

| **S4 Table. Knowledge^a^ and attitude^b^ scores for demographics and influenza vaccination status, healthcare workers, Honduras, 2018 (n=947)** | | | | |
| --- | --- | --- | --- | --- |
| Characteristic | Knowledge score  Mean (SD) | *P*-value^c^ | Attitude score  Mean (SD) | *P*-value^c^ |
| Age in years |  | 0.156 |  | 0.246 |
| 20-30 | 5.75 (1.15) |  | 13.68 (2.82) |  |
| 31-40 | 5.90 (1.11) |  | 13.10 (3.79) |  |
| ≥41 | 5.95 (1.21) |  | 13.32 (3.33) |  |
| Sex |  | 0.057 |  | 0.279 |
| Female | 5.87 (1.19) |  | 13.38 (3.27) |  |
| Male | 6.04 (1.10) |  | 13.10 (3.79) |  |
| Education |  | 0.007 |  | 0.426 |
| ≤Middle school | 5.70 (1.34) |  | 12.98 (3.60) |  |
| High School | 5.83 (1.14) |  | 13.27 (3.48) |  |
| University | 5.97 (1.16) |  | 13.42 (3.35) |  |
| Postgraduate, masters, doctorate | 6.11 (1.01) |  | 13.54 (3.07) |  |
| Marital status |  | 0.088 |  | 0.602 |
| Single | 5.86 (1.12) |  | 13.30 (3.31) |  |
| Married | 6.00 (1.14) |  | 13.28 (3.60) |  |
| Accompanied | 5.72 (1.37) |  | 13.63 (2.94) |  |
| Other | 5.87 (1.21) |  | 12.93 (3.34) |  |
| Profession |  | <0.001 |  | 0.050 |
| Doctor | 6.18 (1.04) |  | 13.76 (3.12) |  |
| Nursing professional | 6.19 (1.03) |  | 13.66 (3.19) |  |
| Nursing assistant | 5.73 (1.22) |  | 13.24 (3.40) |  |
| Other | 5.80 (1.22) |  | 12.94 (3.64) |  |
| Years in profession |  | 0.510 |  | 0.095 |
| ≤10 years | 5.88 (1.17) |  | 13.54 (3.26) |  |
| >10 years | 5.93 (1.18) |  | 13.16 (3.48) |  |
| Works in multiple healthcare facilities |  | 0.148 |  | 0.914 |
| Yes | 5.99 (1.17) |  | 13.34 (3.43) |  |
| No | 5.87 (1.18) |  | 13.31 (3.38) |  |
| Number of patients attended per day |  | 0.684 |  | <0.001 |
| ≤10 | 5.83 (1.26) |  | 12.51 (4.11) |  |
| 11-20 | 5.92 (1.18) |  | 13.49 (3.08) |  |
| 21-30 | 5.95 (1.14) |  | 13.47 (3.42) |  |
| >30 | 5.93 (1.12) |  | 13.76 (2.78) |  |
| Service network |  | 0.705 |  | 0.382 |
| Central | 5.87 (1.22) |  | 13.36 (3.32) |  |
| North | 5.91 (1.14) |  | 13.20 (3.59) |  |
| West | 6.05 (1.15) |  | 13.91 (2.29) |  |
| South | 5.96 (1.11) |  | 12.98 (3.83) |  |
| Health system |  | 0.323 |  | 0.433 |
| Ministry of Health of Honduras | 5.92 (1.18) |  | 13.35 (3.35) |  |
| Honduran Social Security Institute | 5.82 (1.13) |  | 13.12 (3.63) |  |
| Self-reported influenza vaccination in 2017 (n=935)^d^ |  | 0.294 |  | <0.001 |
| Yes | 5.95 (1.20) |  | 14.34 (2.12) |  |
| No | 5.86 (1.09) |  | 11.18 (4.45) |  |
| Self-reported influenza vaccination in 2018 (n=945)^e^ |  | 0.286 |  | <0.001 |
| Yes | 5.95 (1.21) |  | 14.35 (2.19) |  |
| No | 5.87 (1.14) |  | 12.19 (4.05) |  |
| ^a^ Knowledge score was derived from principal components analysis and included: knowledge that influenza may be transmitted from birds/pigs to people, people may contract influenza multiple times, influenza may be spread via contaminated hands, and healthcare workers may transmit influenza to patients; range: 0-7 | | | | |
| ^b^ Attitudes score was derived from principal components analysis and included: belief that vaccination is effective at preventing influenza, lowers risk of hospitalization/death, decreases days of illness, and protects patients; healthcare personnel should get vaccinated every year; would get vaccinated if offered vaccine at home or at work; and recommends vaccination to family and friends; range: 0-16 | | | | |
| ^c^ P-value from t-test or analysis of variance | | | | |
| ^d^ Excluded 12 who did not know vaccination status | | | | |
| ^e^ Excluded 2 who did not know vaccination status | | | | |
