# Supplementary material for: Knowledge, attitudes, and practices of seasonal influenza vaccination in healthcare workers, Honduras
Source: PLoS One. 2021 Feb 4;16(2):e0246379. doi: 10.1371/journal.pone.0246379 (PMC7861374; doi:10.1371/journal.pone.0246379)
Supplement: S5 Table — (DOCX) [file pone.0246379.s005.docx]

| **S5 Table. Knowledge^a^ and attitude^b^ scores for sources of information about influenza vaccination, healthcare workers, Honduras, 2018 (n=947)** | | | | |
| --- | --- | --- | --- | --- |
| Source of information^c^ | Knowledge score  Mean (SD) | *P*-value^d^ | Attitude score  Mean (SD) | *P*-value^d^ |
| Family or friends | 5.91 (1.28) | 0.965 | 13.61 (3.23) | 0.484 |
| Coworkers or peers | 5.94 (1.12) | 0.570 | 13.47 (3.42) | 0.410 |
| Information informally provided in healthcare facility | 5.90 (1.13) | 0.855 | 13.66 (3.05) | 0.013 |
| Training in healthcare facility | 6.09 (1.07) | 0.006 | 14.54 (1.83) | <0.001 |
| Doctor or nurse at healthcare facility | 5.85 (1.16) | 0.573 | 13.58 (3.17) | 0.328 |
| Medical consultation | 5.67 (1.21) | 0.301 | 13.58 (2.91) | 0.690 |
| Brochures or posters | 5.76 (1.00) | 0.453 | 13.08 (2.89) | 0.684 |
| Vocational training | 5.98 (0.67) | 0.871 | 14.84 (1.68) | 0.233 |
| Self-taught | 6.04 (1.15) | 0.006 | 13.50 (3.31) | 0.203 |
| From mass media | 5.97 (1.08) | 0.236 | 12.91 (3.75) | 0.023 |
| ^a^ Knowledge score was derived from principal components analysis and included: knowledge that influenza may be transmitted from birds/pigs to people, people may contract influenza multiple times, influenza may be spread via contaminated hands, and healthcare workers may transmit influenza to patients; range: 0-7 | | | | |
| ^b^ Attitude score was derived from principal components analysis and included: belief that vaccination is effective at preventing influenza, lowers risk of hospitalization/death, decreases days of illness, and protects patients; healthcare personnel should get vaccinated every year; would get vaccinated if offered vaccine at home or at work; and recommends vaccination to family and friends; range: 0-16 | | | | |
| ^c^ Reference: not a source of information | | | | |
| ^d^ *P*-value from t-test | | | | |
