# Supplementary material for: Knowledge, attitudes, and practices of seasonal influenza vaccination in healthcare workers, Honduras
Source: PLoS One. 2021 Feb 4;16(2):e0246379. doi: 10.1371/journal.pone.0246379 (PMC7861374; doi:10.1371/journal.pone.0246379)
Supplement: S6 Table — (DOCX) [file pone.0246379.s006.docx]

| **S6 Table. Associations between demographics and sources of information, and knowledge^a^ and attitude^b^ scores, healthcare workers, Honduras, 2018** | | | | | | |
| --- | --- | --- | --- | --- | --- | --- |
|  | Knowledge score (n= 947) | | | Attitude score (n= 935)^c^ | | |
| Variable | β^d^ | SE | *P*-value | β^d^ | SE | *P*-value |
| Profession (Ref: doctor) |  |  | <0.001 |  |  | – |
| Nursing professional | -0.01 | 0.13 |  | – | – |  |
| Nursing assistant | -0.44 | 0.10 |  | – | – |  |
| Other | -0.37 | 0.11 |  | – | – |  |
| ≤10 years in profession (Ref: >10 years) | – | – | – | 0.35 | 0.20 | 0.077 |
| Number of patients attended per day (Ref: ≤10) |  |  | – |  |  | 0.001 |
| 11-20 | – | – |  | 0.89 | 0.28 |  |
| 21-30 | – | – |  | 0.61 | 0.29 |  |
| >30 | – | – |  | 0.99 | 0.27 |  |
| Source of information (Ref: no) |  |  |  |  |  |  |
| Informal information in healthcare facility | – | – | – | 0.44 | 0.20 | 0.030 |
| Training in healthcare facility | 0.20 | 0.09 | 0.030 | 1.11 | 0.24 | <0.001 |
| Mass media | – | – | – | -0.31 | 0.22 | 0.146 |
| Vaccinated for influenza in previous year (Ref: no) | – | – | – | 2.97 | 0.21 | <0.001 |
| Knowledge score (1-unit increase) | – | – | – | 0.13 | 0.08 | 0.117 |
| SE: standard error | | | | | | |
| ^a^ Knowledge score was derived from principal components analysis and included: knowledge that influenza may be transmitted from birds/pigs to people, people may contract influenza multiple times, influenza may be spread via contaminated hands, and healthcare workers may transmit influenza to patients; range: 0-7 | | | | | | |
| ^b^ Attitude score was derived from principal components analysis and included: belief that vaccination is effective at preventing influenza, lowers risk of hospitalization/death, decreases days of illness, and protects patients; healthcare personnel should get vaccinated every year; would get vaccinated if offered vaccine at home or at work; and recommends vaccination to family and friends; range: 0-16 | | | | | | |
| ^c^ Excluded 12 participants who did not know vaccination status in 2017 | | | | | | |
| ^d^ Adjusted for the other variables listed in the model | | | | | | |
