# Supplementary material for: Knowledge, attitudes, and practices of seasonal influenza vaccination in healthcare workers, Honduras
Source: PLoS One. 2021 Feb 4;16(2):e0246379. doi: 10.1371/journal.pone.0246379 (PMC7861374; doi:10.1371/journal.pone.0246379)
Supplement: S7 Table — (DOCX) [file pone.0246379.s007.docx]

| **S7 Table. Clinical manifestations seven days after vaccination, healthcare workers (n = 491), Honduras, 2018** | | |
| --- | --- | --- |
| Clinical manifestation | n | % (95% CI) |
| Vaccination site pain | 89 | 18.1 (14.7–21.5) |
| Flu-like symptoms | 84 | 17.1 (13.8–20.5) |
| General discomfort | 64 | 13.0 (10.0–16.0) |
| Fever | 47 | 9.6 (6.9–12.2) |
| Urticaria | 10 | 2.0 (0.8–3.3) |
| Inflammation at the vaccination site | 8 | 1.6 (0.5–2.8) |
| Dizziness | 6 | 1.2 (0.2–2.2) |
| Hematoma at the vaccination site | 1 | 0.2 (0–0.6) |
| CI: confidence interval | | |
