# Supplementary material for: Knowledge, attitudes, and practices of seasonal influenza vaccination in healthcare workers, Honduras
Source: PLoS One. 2021 Feb 4;16(2):e0246379. doi: 10.1371/journal.pone.0246379 (PMC7861374; doi:10.1371/journal.pone.0246379)
Supplement: S1 Fig — This figure excluded two healthcare workers who did not know their vaccination status. (DOCX) [file pone.0246379.s008.docx]

**S1 Fig. Seasonal influenza vaccination coverage among 945 healthcare workers, and proportion who would get vaccinated if offered the vaccine at work by hospital, Honduras, 2018.** This figure excluded two healthcare workers who did not know their vaccination status.
