# Supplementary material for: Knowledge, attitudes, and practices of seasonal influenza vaccination in healthcare workers, Honduras
Source: PLoS One. 2021 Feb 4;16(2):e0246379. doi: 10.1371/journal.pone.0246379 (PMC7861374; doi:10.1371/journal.pone.0246379)
Supplement: S1 Questionnaire — (DOCX) [file pone.0246379.s009.docx]

**Encuesta a personal de salud**

[*Nota: los textos en color* ***celeste*** *estarán ocultos para los encuestados*]

***Datos demográficos de la persona entrevistada***

1. Código para el estudio: ___________
2. Nombre del establecimiento: ____________________________________
3. Nombre de la persona entrevistada: ____________________________________
4. Edad: ________ años.
5. Sexo:  Masculino;  Femenino
6. Estado civil (marque solo una)

Soltero(a);  Casado(a) o en unión libre;  Divorciado(a);  Viudo(a);  No responde

1. ¿Hasta qué nivel de estudios ha llegado usted? (marque solo una):
2. No sabe leer ni escribir, y no ha realizado estudios formales
3. Sabe leer y escribir, pero no ha realizado estudios formales
4. Primaria incompleta
5. Primaria completa
6. Secundaria incompleta
7. Secundaria completa
8. Bachillerato o diversificado incompleto
9. Bachillerato o diversificado completo
10. Estudios universitarios
11. Maestría profesional, especialización o postgrado
12. Doctorado
13. No responde
14. Profesión (marque solo una):
15. Médico especialista (Cuál especialidad:__________________)
16. Médico general
17. Profesional en enfermería
18. Profesional en laboratorio (Químico Biólogo)
19. Profesional de servicio de apoyo (Cuál profesión:______________)
20. Profesional odontología.
21. Auxiliar en enfermería
22. Técnico laboratorio
23. Técnico o auxiliar en odontología
24. Técnico o asistente en servicio de apoyo (Cuál servicio:_______________)
25. Otro ¿Cuál? _________________________________________________
26. Servicio dónde labora (puede marcar más de una):

Atención de niños

Atención de adultos

Sala de operaciones

Cuidados intensivos

Laboratorio

Radiología

Servicios de apoyo

Unidad de Emergencias

Otro (Cuál:___________________________________________________________)

1. ¿Cuántos años en ejercicio de su profesión tiene usted?: _______ años.
2. ¿Cuántos años tiene usted laborando en esta institución?: ________años.
3. ¿Cuál es el promedio de pacientes que usted atiende por día?: __________ pacientes.

No aplica

1. Además de trabajar aquí ¿Trabaja en otros establecimientos?

Sí;  No;  No sabe  No responde

Si es Sí:

1. Total de horas que brinda atención en este y otros establecimientos: _______Horas

**Datos del otro establecimiento (1)**

1. Nombre del establecimiento: ____________________________________

Público  Seguridad Social  Privado  Otro tipo (Cuál: _________________)

**Datos del otro establecimiento (2)**

1. Nombre del establecimiento: ____________________________________

Público  Seguridad Social  Privado  Otro tipo (Cuál: _________________)

**En la siguiente sección le leeremos una serie de aseveraciones sobre la influenza y la vacunación contra la misma. Le solicitamos que a cada aseveración anote si está de acuerdo o en desacuerdo. También puede decirnos que no sabe al respecto, o que no desea comentar al respecto.**

***Conocimientos sobre la transmisión de la influenza***

1. Las personas pueden diseminar el virus de la influenza aunque se sientan bien.

(Responder en escala del 1 al 5. Solamente puede escoger una opción)

1 = totalmente en desacuerdo,

2 = ligeramente en desacuerdo

3 = ligeramente de acuerdo

4 = totalmente de acuerdo

No sabe

No responde

1. La influenza puede ser transmitida de las aves o los cerdos a las personas

(Responder en escala del 1 al 5. Solamente puede escoger una opción)

1 = totalmente en desacuerdo,

2 = ligeramente en desacuerdo

3 = ligeramente de acuerdo

4 = totalmente de acuerdo

No sabe

No responde

1. Una persona puede adquirir la influenza aunque haya tenido una infección previa por virus de la influenza.

(Responder en escala del 1 al 5. Solamente puede escoger una opción)

1 = totalmente en desacuerdo,

2 = ligeramente en desacuerdo

3 = ligeramente de acuerdo

4 = totalmente de acuerdo

No sabe

No responde

1. Las personas pueden transmitir la influenza únicamente después de que los síntomas aparezcan.

(Responder en escala del 1 al 5. Solamente puede escoger una opción)

1 = totalmente en desacuerdo,

2 = ligeramente en desacuerdo

3 = ligeramente de acuerdo

4 = totalmente de acuerdo

No sabe

No responde

1. Las personas pueden adquirir la influenza si toca su boca o nariz después de haber tocado algo contaminado con el virus de la influenza.

(Responder en escala del 1 al 5. Solamente puede escoger una opción)

1 = totalmente en desacuerdo,

2 = ligeramente en desacuerdo

3 = ligeramente de acuerdo

4 = totalmente de acuerdo

No sabe

No responde

1. Es probable que un trabajador de salud transmita la influenza a los pacientes que atiende.

(Responder en escala del 1 al 5. Solamente puede escoger una opción)

1 = totalmente en desacuerdo,

2 = ligeramente en desacuerdo

3 = ligeramente de acuerdo

4 = totalmente de acuerdo

No sabe

No responde

**A continuación le haremos unas preguntas sobre la vacuna contra la influenza estacional**

***Información sobre la vacuna contra la influenza estacional***

1. Considero que he recibido toda la información que necesito para decidir si me vacuno contra la influenza estacional.

(Responder en escala del 1 al 4. Solamente puede escoger una opción)

1 = totalmente en desacuerdo,

2 = ligeramente en desacuerdo

3 = ligeramente de acuerdo

4 = totalmente de acuerdo

No sabe

No responde

1. De las siguientes opciones: ¿De dónde obtuvo la información sobre la vacuna contra la influenza estacional? (puede escoger más de una)
2. De conversaciones con familiares o amigos.
3. De conversaciones con compañeros de trabajo (pares).
4. De los medios masivos de comunicación.

¿De los siguientes medios masivos de comunicación cuales Cuáles medios de comunicación?

|  | **Confío altamente** | **Confío** | **No confío** | **No contestó** |
| --- | --- | --- | --- | --- |
| c.1  Redes sociales como Facebook, Twitter, u otra |  |  |  |  |
| c.2  Radio |  |  |  |  |
| c.3  Televisión |  |  |  |  |
| c.4  Periódicos |  |  |  |  |
| c.5  Seguros médicos |  |  |  |  |
| c.6  Asociaciones profesionales médicas |  |  |  |  |
| c.7  OPS/OMS |  |  |  |  |
| c.8  CDC |  |  |  |  |
| c.9  Compañías farmacéuticas |  |  |  |  |
| c.10  Otro c.11 ¿Cuál? ___________________________ |  |  |  |  |
| c.12  Otro c.13 ¿Cuál? ___________________________ |  |  |  |  |
| c.14  Otro c.15 ¿Cuál? ___________________________ |  |  |  |  |

1. Autodidacta

¿Cuáles fuentes de información utiliza de manera autodidacta?

d.1  Literatura científica publicada en internet

d.2  Libros de medicina

d.3  Revistas científicas impresas

d.4  mi experiencia adquirida en la clínica

d.5  Otras

d.5 ¿Cuál? __________________________________

1. De información proporcionada en el establecimiento de salud informalmente.
2. Durante una capacitación en el establecimiento de salud.
3. De un médico o enfermera del establecimiento de salud donde labora.
4. En una consulta médica.
5. Otra. ¿Cuál? _______________________________________

**Por favor denos su opinión las siguientes aseveraciones (escoger una opción por pregunta):**

***Beneficios percibidos y efectividad de la acción***

1. La vacuna contra la influenza estacional es efectiva para prevenir la enfermedad

(Responder en escala del 1 al 4. Solamente puede escoger una opción)

1 = totalmente en desacuerdo,

2 = ligeramente en desacuerdo

3 = ligeramente de acuerdo

4 = totalmente de acuerdo

No sabe

No responde

1. La vacuna contra la influenza estacional puede disminuir el riesgo de hospitalizaciones y muertes asociadas con el virus de la influenza.

(Responder en escala del 1 al 4. Solamente puede escoger una opción)

1 = totalmente en desacuerdo,

2 = ligeramente en desacuerdo

3 = ligeramente de acuerdo

4 = totalmente de acuerdo

No sabe

No responde

1. La vacuna contra la influenza estacional puede disminuir los días de enfermedad por el virus de la influenza.

(Responder en escala del 1 al 4. Solamente puede escoger una opción)

1 = totalmente en desacuerdo,

2 = ligeramente en desacuerdo

3 = ligeramente de acuerdo

4 = totalmente de acuerdo

No sabe

No responde

1. La vacunación del personal de salud contra la influenza estacional puede contribuir proteger a los pacientes en el establecimiento de salud.

(Responder en escala del 1 al 4. Solamente puede escoger una opción)

1 = totalmente en desacuerdo,

2 = ligeramente en desacuerdo

3 = ligeramente de acuerdo

4 = totalmente de acuerdo

No sabe

No responde

1. ¿El personal de salud debe vacunarse cada año contra la influenza?

Sí,  No  No sabe  No responde

**Por favor responda las siguientes preguntas**

***Actitud hacia la vacunación contra la influenza***

1. Si personal de salud le visitara en su lugar de trabajo, y usted tuviera tiempo disponible: ¿Permitiría que le vacunasen a usted contra la influenza?

Sí,  No  No sabe  No responde

1. Si hubiera una jornada de vacunación y el personal de salud le visitara en su hogar ¿Permitiría que sus familiares fueran vacunados contra la influenza?

Sí,  No  No sabe  No responde

1. ¿Usted les recomendaría a sus familiares, amigos, vecinos, compañeros de estudio o trabajo que se vacunaren contra la influenza?

Sí,  No  No sabe  No responde

***Vacunación previa***

1. ¿Fue usted fue vacunado(a) contra la influenza en la campaña de vacunación del año 2017?

Sí  No  Planee vacunarme, pero no pude  No sabe  No responde

***Voluntad para vacunarse***

1. ¿Piensa usted vacunarse contra la influenza en la actual campaña de vacunación del 2018?

Sí  No  No sabe  No responde

Si es No,

1. De las siguientes opciones, ¿Cuál o cuáles son los motivos por lo que no se vacunará en la campaña de 2018? (puede marcarse más de una) [*esta pregunta se hará en caso que aun no haya finalizado la campaña de vacunación en el establecimiento de salud*]

***Rechazo por temor a efectos adversos***

Tiene temor a enfermar de gripe si se vacuna contra la influenza estacional

Tiene temor de los efectos secundarios

Tiene temor al dolor que causa la inyección

Estaba embarazada *(esta opción debería ocultarse si el sexo es Masculino)*

Estaba dando lactancia materna *(esta opción debería ocultarse si el sexo es Masculino)*

***Percepción de falta de utilidad de la vacunación***

Considera que la vacuna no es efectiva

No cree que pueda enfermar gravemente por influenza (no vale la pena vacunarse)

No cree que el que usted se vacune evite que sus compañeros en este establecimiento enfermen por influenza

No estoy en contacto con pacientes con factores de riesgo para complicaciones (Ejemplo: niños, adultos mayores, personas con enfermedades crónicas, o mujeres embarazadas)

No confía en las vacunas que actualmente están en uso

No confía en la cadena de frío para la conservación de las vacunas

***Limitaciones en el acceso a la vacuna***

Nadie le dijo que debía vacunarse contra la influenza

No se le ha ofrecido la vacuna

No sabe a dónde acudir por una vacuna

No tiene tiempo para ir a vacunarse

Mi jefe / supervisor no me dio permiso para ir a vacunarme

La vacuna es demasiado cara

***Influencia social de compañeros de trabajo/familiares/amigos***

La vacunación contra la influenza no es aceptada por los demás compañeros en el establecimiento de salud

Sus familiares le dijeron que no se vacunara

Sus amigos le dijeron que no se vacunara

Otro ¿Cuál?_______________________________________________________

1. ¿Fue usted fue vacunado(a) contra la influenza en la campaña de vacunación del año 2018?

Sí  No  No sabe  No responde (*si es Sí pase a la pregunta 35*)

Si es No,

1. De las siguientes opciones, ¿Cuál o cuáles fueron los motivos por lo que no se vacunó en la campaña de 2018? (puede marcarse más de una) [*esta pregunta se hará en caso que haya finalizado la campaña de vacunación en el establecimiento de salud*]

***Rechazo por temor a efectos adversos***

Tiene temor a enfermar de gripe si se vacuna contra la influenza estacional

Tiene temor de los efectos secundarios

Tiene temor al dolor que causa la inyección

Estaba embarazada *(esta opción debería ocultarse si el sexo es Masculino)*

Estaba dando lactancia materna *(esta opción debería ocultarse si el sexo es Masculino)*

***Percepción de falta de utilidad de la vacunación***

Considera que la vacuna no es efectiva

No cree que pueda enfermar gravemente por influenza (no vale la pena vacunarse)

No cree que el que usted se vacune evite que sus compañeros en este establecimiento enfermen por influenza

No estoy en contacto con pacientes con factores de riesgo para complicaciones (Ejemplo: niños, adultos mayores, personas con enfermedades crónicas, o mujeres embarazadas)

No confía en las vacunas que actualmente están en uso

No confía en la cadena de frío para la conservación de las vacunas

***Limitaciones en el acceso a la vacuna***

Nadie le dijo que debía vacunarse contra la influenza

No se le ha ofrecido la vacuna

No sabe a dónde acudir por una vacuna

No tiene tiempo para ir a vacunarse

Mi jefe / supervisor no me dio permiso para ir a vacunarme

La vacuna es demasiado cara

***Influencia social de compañeros de trabajo/familiares/amigos***

La vacunación contra la influenza no es aceptada por los demás compañeros en el establecimiento de salud

Sus familiares le dijeron que no se vacunara

Sus amigos le dijeron que no se vacunara

Otro ¿Cuál?_______________________________________________________

1. ¿Presentó algún síntoma en los 7 días posteriores a la vacunación del 2018?

Sí  No  No sabe  No responde

Si es Sí:

1. ¿Cuál síntoma presentó?

Malestar general;

Dolor en el sitio de la vacunación;

Hinchazón y/o enrojecimiento en el sitio de vacunación

Hematoma en el sitio de vacunación

Urticaria

Reacción alérgica

Fiebre o sensación de fiebre;

Mareos

Síntomas similares a una gripe;

Otro. ¿Cuál? ___________________

No sabe  No responde

***Conocimientos sobre la composición de la vacuna y la capacidad de que esta ocasione la enfermedad***

1. En Honduras, La vacuna de la influenza estacional que utiliza la Secretaría de Salud y el Instituto Hondureño de Seguridad Social está compuesta por: (por favor marque todas las que apliquen)

Virus vivos atenuados

Virus inactivados (segmentos de virus muertos)

No sabe  No responde

1. ¿La vacuna de la influenza puede causar síntomas similares a los de una gripe?

Sí,  No  No sabe  No responde

**Muchas gracias por haber participado en esta encuesta**

**Survey in English**

1. Code assigned to the participant: ___________
2. Healthcare facility name: ____________________________________
3. Name of the interviewee: ____________________________________
4. Age: ________ years.
5. Sex:  Male;  Female
6. Marital status (check one)

Single (never married or accompanied)

Married

Accompanied

Divorced

Separated

Widow

No response

1. To what level of education have you come? (Check one):

Cannot read or write; no formal education

Literate, but has no formal education

Incomplete primary

Full primary

Incomplete secondary

Completed secondary

Diversified secondary education or incomplete

Diversified secondary education or full

University

Professional expertise, specialization or postgraduate

Doctorate

No response

1. Profession (check one):

Specialist (P8A) (What specialty: __________________)

General practitioner

Professional Nursing

Professional Laboratory (biochemist)

Professional Support Service (P8B) (What profession: ______________)

Professional dentistry

Nursing Assistant

Technical laboratory

Technical or auxiliary dental

Technical or service support assistant (P8C) (What service: _____________)

Other (p8d) What? ________________________________________________

1. Service where work (you can check more than one):
2. Child care
3. Adult care
4. Operations room
5. Intensive care
6. Laboratory
7. Radiology
8. Supporting services
9. Emergency Unit
10. Other (p9i1) (What: ________________________________________)
11. How many years in exercising the profession do you have ?: _______ years.
12. How many years have you been working in this institution ?: ________ years.
13. What is the average number of patients per day you attend ?: __________ patients.
14. In addition to working here, do you work in other establishments?

Yes;  No;  Does not know  no response

If yes:

1. Total hours providing care in this and other establishments: _______Hours

**Details of other establishment (1)**

1. Healthcare facility name: ____________________________________

Public  Social Security  Private  Other (p15a) (What: _________________)

**Details of other establishment (2)**

1. Healthcare facility name: ____________________________________

Public  Social Security  Private  Other (P16a) (What: _________________)

**In the following section we will read a series of assertions on influenza and its vaccination. We ask that each statement note whether you agree or disagree. You can also tell us not to know about it, or do not want to respond.**

1. People may spread influenza even without symptoms.

(Answer on a scale of 1 to 5. You can only choose one)

1 = strongly disagree,

2 = slightly disagree

3 = slightly according

4 = totally agree

Does not know

no response

1. Influenza may be transmitted from birds or pigs to people

(Answer on a scale of 1 to 5. You can only choose one)

1 = strongly disagree,

2 = slightly disagree

3 = slightly according

4 = totally agree

Does not know

no response

1. People may contract influenza even if they have previously contracted influenza.

(Answer on a scale of 1 to 5. You can only choose one)

1 = strongly disagree,

2 = slightly disagree

3 = slightly according

4 = totally agree

Does not know

no response

1. People can spread flu only after symptoms appear.

(Answer on a scale of 1 to 5. You can only choose one)

1 = strongly disagree,

2 = slightly disagree

3 = slightly according

4 = totally agree

Does not know

no response

1. Influenza may be spread by touching one's mouth or nose with contaminated hands.

(Answer on a scale of 1 to 5. You can only choose one)

1 = strongly disagree,

2 = slightly disagree

3 = slightly according

4 = totally agree

Does not know

no response

1. Healthcare workers may transmit influenza to their patients.

(Answer on a scale of 1 to 5. You can only choose one)

1 = strongly disagree,

2 = slightly disagree

3 = slightly according

4 = totally agree

Does not know

no response

**Now we will ask you about the seasonal influenza vaccine**

1. Received adequate information to decide whether to get vaccinated.

(Answer on a scale of 1 to 4. You can only choose one)

1 = strongly disagree,

2 = slightly disagree

3 = slightly according

4 = totally agree

Does not know

no response

1. Sources of information about influenza vaccination (You can choose more than one)
2. Conversations with family or friends.
3. Conversations with colleagues (peers).
4. The mass media.

Of the following which mass media: Which media?

|  | **Fully trust** | **Partially trust** | **Do not trust** | **No response** |
| --- | --- | --- | --- | --- |
| 1. Social networks like Facebook, Twitter, or other |  |  |  |  |
| 1. Radio |  |  |  |  |
| 1. TV |  |  |  |  |
| 1. Newspapers |  |  |  |  |
| 1. Medical insurance |  |  |  |  |
| 1. Professional medical associations |  |  |  |  |
| 1. PAHO / WHO |  |  |  |  |
| 1. CDC |  |  |  |  |
| 1. Pharmaceutical companies |  |  |  |  |
| 1. Other p21c10a What? ___________________________ |  |  |  |  |
| 1. Another p21c11a Which? ___________________________ |  |  |  |  |
| 1. Another p21c12a Which? ___________________________ |  |  |  |  |

1. Self-teaching

What sources used for self-teaching?

1. Scientific literature published online
2. Medical books
3. Printed journals
4. My experience at the clinic
5. Other (p24d5a) What? ______________________________
6. Information provided in the health facility informally.
7. During training in the health facility.
8. A doctor or nurse at the health facility where he works.
9. In a medical consultation.
10. Other. (P24i1) What? _______________________________________

**Please give us your opinion the following statements (choose one option per question):**

1. The vaccine is effective at preventing influenza

(Answer on a scale of 1 to 4. You can only choose one)

1 = strongly disagree,

2 = slightly disagree

3 = slightly according

4 = totally agree

Does not know

no response

1. The vaccine lowers the risk of hospitalization and death

(Answer on a scale of 1 to 4. You can only choose one)

1 = strongly disagree,

2 = slightly disagree

3 = slightly according

4 = totally agree

Does not know

no response

1. The vaccine may decrease the days of illness from influenza

(Answer on a scale of 1 to 4. You can only choose one)

1 = strongly disagree,

2 = slightly disagree

3 = slightly according

4 = totally agree

Does not know

no response

1. Vaccinating healthcare personnel protects patients

(Answer on a scale of 1 to 4. You can only choose one)

1 = strongly disagree,

2 = slightly disagree

3 = slightly according

4 = totally agree

Does not know

no response

1. Healthcare personnel should get vaccinated for influenza every year

Yes,  No  Does not know  no response

**Please answer the following questions**

1. Would you get vaccinated for influenza if offered the vaccine at work?

Yes,  No  Does not know  no response

1. Would you get vaccinated for influenza if offered the vaccine at home?

Yes,  No  Does not know  no response

1. Do you recommend the influenza vaccine to family and friends?

Yes,  No  Does not know  no response

1. Were you vaccinated for seasonal influenza in 2017?

Yes  No  Plan vaccinated, but could not  Does not know  no response

1. Do you planning to get the flu-shot during the current vaccination campaign in 2018?

Yes  No  Does not know  no response

It is, is not it,

1. Of the following options, what reasons do you have to avoid the flu-shot during 2018 vaccination campaign? (Can mark more than one)
2. Fear of contracting influenza
3. Fear of side effects
4. Fear of injection pain
5. I was pregnant (this option should be hidden if sex is male)
6. Was breastfeeding
7. Believes that the vaccine is not effective
8. Influenza does not cause serious illness
9. The vaccine does not prevent colleagues from contracting influenza
10. Not in contact with patients who have influenza
11. No confidence in current vaccines
12. No confidence in cold-chain for preserving vaccines
13. Not informed to get vaccinated
14. Was not offered the vaccine
15. Unaware of where to get vaccinated
16. Too busy to get vaccinated
17. Boss did not give permission to get vaccinated
18. The vaccine is too expensive
19. Vaccine not accepted by peers
20. Relatives said not to get vaccinated
21. Friends said not to get vaccinated
22. Other (p35u1) What? ________________________________________________
23. Were you vaccinated during 2018 influenza vaccination campaign?

Yes  No  Does not know  no response (If yes, go to question 37)

It is, is not it,

1. Of the following options, what or what were the reasons soyou was not vaccinated during the campaign of 2018? (Can be marked more than one) [this question will be if you have completed the vaccination campaign in the health facility]
2. Fear of contracting influenza
3. Fear of side effects
4. Fear of injection pain
5. I was pregnant
6. Was breastfeeding
7. Believes the vaccine is not effective
8. Influenza does not cause serious illness
9. The vaccine does not prevent colleagues from contracting influenza
10. Not in contact with patients who have influenza
11. No confidence in current vaccines
12. No confidence in cold-chain for preserving vaccines
13. Not informed to get vaccinated
14. Was not offered the vaccine
15. Unaware of where to get vaccinated
16. Too busy to get vaccinated
17. Boss did not give permission to get vaccinated
18. The vaccine is too expensive
19. Vaccine not accepted by peers
20. Relatives said not to get vaccinated
21. Friends said not to get vaccinated
22. Other (p37u1) What? _______________________________________________
23. Did you file any symptoms within 7 days after vaccination in 2018?

Yes  No  Does not know  no response

If yes:

1. What symptom presented?
2. General discomfort;
3. Pain at the site of vaccination;
4. Inflammation at the site of vaccination
5. Hematoma at the site of vaccination
6. Urticaria
7. Allergic reaction
8. Fever;
9. Dizziness
10. flu-like symptoms;
11. Other. (P40j1) What? ___________________
12. Does not know
13. no response
14. In Honduras, the seasonal influenza vaccine that uses the Ministry of Health and the Honduran Social Security Institute it is composed of: (please check all that apply)
15. live attenuated
16. Inactivated (killed virus segments)
17. Does not know
18. no response
19. Does the influenza vaccine cause flu-like symptoms?

Yes,  No  Does not know  no response

**Thank you very much for participating in this survey**
