# Supplementary material for: Knowledge, attitudes, and practices of seasonal influenza vaccination in healthcare workers, Honduras
Source: PLoS One. 2021 Feb 4;16(2):e0246379. doi: 10.1371/journal.pone.0246379 (PMC7861374; doi:10.1371/journal.pone.0246379)
Supplement: S1 File — (DOCX) [file pone.0246379.s010.docx]

**S1 File: Equation used to obtain sample sizes for surveys of healthcare workers.**

Schaeffer R, Mendenhall W, Ott L. Elementary Survey Sampling, Boston. Massachusetts: PWS-Kent Publishing Company. 1990

$$\boldsymbol{n=deff\times}\frac{\boldsymbol{N}\boldsymbol{Z}_{\boldsymbol{1-\alpha/2}}^{\boldsymbol{2}}\boldsymbol{P(1-P)}}{\left( \boldsymbol{N-1} \right)\boldsymbol{d}^{\boldsymbol{2}}\boldsymbol{+}\boldsymbol{Z}_{\boldsymbol{1-\alpha/2}}^{\boldsymbol{2}}\boldsymbol{P(1-P)}}$$

Where

*N* = population size, $Z_{1-\alpha/2}$ = Quantile of a variable with standard normal distribution

$100(1-\alpha)\%$= Confidence level

*P* = Expected proportion in the population.

*d* = Absolute accuracy required

$deff=$ Design effect
